# Diagnosis and treatment of perforated or bleeding peptic ulcers: 2013 WSES position paper

**DOI:** 10.1186/1749-7922-9-45

**Published:** 2014-08-03

**Authors:** Salomone Di Saverio, Marco Bassi, Nazareno Smerieri, Michele Masetti, Francesco Ferrara, Carlo Fabbri, Luca Ansaloni, Stefania Ghersi, Matteo Serenari, Federico Coccolini, Noel Naidoo, Massimo Sartelli, Gregorio Tugnoli, Fausto Catena, Vincenzo Cennamo, Elio Jovine

**Affiliations:** 1 Emergency and General Surgery Dept, Maggiore Hospital– Bologna Local Health District, Bologna, Italy; 2 Emergency and Trauma Surgery Dept., Maggiore Hospital of Parma, Parma, Italy; 3 General and Emergency and Trauma Surgery, I unit, Ospedali Riuniti, Bergamo, Italy; 4 Port Shepstone Regional Hospital, Port Shepstone, South Africa - Nelson R Mandela School of Medicine, University of KwaZulu-Natal, Durban, South Africa; 5 Department of Surgery, Hospital of Macerata, Macerata, Italy; 6 Liver and Multivisceral Transplantation Unit, University of Modena&Reggio Emilia – Policlinico Hospital, Modena, Italy; 7 Department of Gastroenterology and Operative Endoscopy, Maggiore Hospital– Bologna Local Health District, Bologna, Italy

## Diagnosis and treatment of perforated peptic ulcer (Dr. S. Di Saverio MD)

### Introduction

Every year peptic ulcer disease (PUD) affects 4 milion people around the world [1]. Complications are encountered in 10%-20% of these patients and 2%-14% of the ulcers will perforate [2,3]. Perforated peptic ulcer (PPU) is relatively rare, but life-threatening with the mortality varying from 10% to 40% [2,4-6]. More than half of the cases are female and they are usually older and have more comorbidities than their male counterparts [6]. Main etiologic factors include use of non-steroidal anti-inflammatory drugs (NSAIDs), steroids, smoking, Helicobacter pylori and a diet high in salt [3,7]. All these factors have in common that they affect acid secretion in the gastric mucosa. Defining the exact etiological factor in any given patient may often be difficult, as more than one risk factor may be present and they tend to interact [8]. While previous reports have shown a seasonal variation in the incidence of PPU, others have failed to find such a pattern [9-11]. Other causes of gastroduodenal perforation are traumatic, neoplastic, foreign body or corrosive ingestion, and those that occur as a result of a diagnostic or therapeutic intervention (iatrogenic). Traumatic injury to the stomach and duodenum causing perforation is rare, comprising only 5.3% of all blunt hollow viscus organ injuries, but is associated with a complication rate of 27% to 28% [12]. Perforations from malignancy can result from obstruction and increased luminal pressure, or from successful treatment and response to chemotherapy and involution of a previously transmural tumor [13]. Foreign bodies, ingested either intentionally or accidentally can cause perforations, either through direct injury or as a result of luminal obstruction [14,15] (Table 1).

**Table 1:** **Causes of gastro**-**duodenal perforation**

**Non-traumatic**	**Traumatic**
Gastric ulcer	Iatrogenic
Duodenal ulcer	Foreign body
Obstruction	Violence
Ischemia	
Malignancy	

Iatrogenic injury is an increasing cause of gastroduodenal perforation. The increasing use of esophagoduodenoscopy for diagnosis and therapy is associated with an increase in procedure-related perforations [16]. Gastroduodenal perforation has also been reported as a complication of a variety of abdominal procedures including Inferior Vena Cava filter placement [17,18], ERCP [19,20], and biliary stents [21].

### Outcomes

When PPU are diagnosed expeditiously and promptly treated, outcomes are excellent. Mortality ranges from 6% to 14% in recent studies [22-24]. Poor outcomes have been associated with increasing age, major medical illness, peri-operative hypotension [25], and delay in diagnosis and management (greater than 24 hours) [26]. With improvements in resuscitation, hypotension may no longer be a significant prognostic indicator [27]. Advanced age (greater than 70 years) is associated with a higher mortality with rates of approximately 41% [28,29]. Several scoring systems including the Boey scoring system [26] (Table 2) and the Mannheim Peritonitis Index (MPI) [30] have been used to stratify the risk of the patients and predict the outcomes of patients with perforated peptic ulcer. The Boey score is the most commonly and easily implemented among these scoring systems, and accurately predicts perioperative morbidity and mortality.

**Table 2:** Boey score and outcomes

**Risk score**	**Mortality (OR)**	**Morbidity (OR)**
1	8% (2.4)	47% (2.9)
2	33% (3.5)	75% (4.3)
3	38% (7.7)	77% (4.9)

Boey score factors.

Concomitant severe medical illness.

Preoperative shock.

Duration of perforation > 24 hours.

Score: 0–3 (Each factor scores 1 point if positive).

Adapted from Lohsiriwat V, Prapasrivorakul S, Lohsiriwat D. Perforated peptic ulcer: clinical presentation, surgical outcomes, and the accuracy of the Boey scoring system in predicting postoperative morbidity and mortality. World J Surg. 2009 Jan;33(1):80–65.

Moller et al. derived the Peptic Ulcer Perforation score (PULP score), a clinical prediction rule for 30-day mortality. The score assesses and compares its prognostic performance with the American Society of Anaesthesiologists (ASA) and Boey scores [31].

Morbidity is common after perforation, with rates ranging from 17% to 63% [32,33]. Pulmonary and wound infections are the most common postoperative infections. Fungal infections after perforation are fairly common (between 13 and 37%) and when identified are associated with significant mortality (up to 21.7%) [34,35]. More recently a study comparing three scoring systems (American Society of Anesthesiologists (ASA), Boey and peptic ulcer perforation (PULP)) regarding the ability to predict mortality in PPU, found that the PULP score had an odds ratio (OR) of 18.6 and the ASA score had an OR of 11.6, both with an area under the curve (AUC) of 0.79. The Boey score had OR of 5.0 and AUC of 0.75. Hypoalbuminaemia alone (≤37 g/l) achieved OR of 8.7 and AUC of 0.78 being the strongest single predictor of mortality [36]. A further new prognostic score has been proposed for perforated duodenal ulcers, including as predictors of poor prognosis factors such as the presence of multiple gut perforations, the size of largest perforation >0.5 cm, amount of peritoneal fluid >1000 ml, simple closure, development of complications, post-operative systemic septicaemia and winter/autumn season of presentation. The new scoring system had an overall sensitivity of 85.12% and specificity of 80.67% [37].

### Diagnosis

Prompt diagnosis of gastroduodenal perforation requires a high index of suspicion based on history and clinical examination. A history of intermittent abdominal pain or gastroesophageal reflux is common. Additionally, known peptic ulcer disease that has been inadequately treated or with ongoing symptoms and sudden exacerbation of pain can be suspicious for perforation. A history of recent trauma or instrumentation followed by abdominal pain and tenderness should alert the clinician to the potential for injury. Patients with gastroduodenal perforation usually present with abdominal pain and peritoneal irritation from leakage of acidic gastric contents. However, physical examination findings may be equivocal, and peritonitis may be minimal or absent, particularly in patients with contained leaks [38]. Patients *in extremis* may also present with altered mental status, further compromising an accurate and reliable physical examination. Laboratory studies are not useful in the acute setting as they tend to be nonspecific, but leukocytosis, metabolic acidosis, and elevated serum amylase may be associated with perforation [38].

Free air under the diaphragm found on an upright chest X-ray is indicative of hollow organ perforation and mandates further work-up and/or exploration. In the setting of an appropriate history and peritonitis on examination, free air on X-ray is sufficient to justify exploration. Patients without pneumoperitoneum at admission on plain chest radiograph, should be evaluated further by computed tomography (CT) scanning with oral contrast.

The increased use of CT scans has greatly improved our ability to detect perforation. Suspicious findings on CT scan include unexplained intraperitoneal fluid, pneumoperitoneum, bowel wall thickening, mesenteric fat streaking, mesenteric hematoma and extravasation of contrast.

However, up to 12% of patients with traumatic perforations may have a normal CT scan. Adding oral contrast and performing triple contrast CT scan may improve diagnostic sensitivity and specifity [39,40].

In the setting of trauma, diagnostic peritoneal lavage (DPL) has essentially been replaced by the focused assessment by sonography for trauma (FAST), which lacks specificity for hollow organ perforation [41,42]. Victims of penetrating trauma with signs of peritonitis require surgical exploration without further diagnostic workup. In blunt trauma patients, and in penetrating trauma patients without peritonitis, in whom the trajectory of the missile may be unclear, CT scanning of the abdomen and pelvis with oral and intravenous contrast remains the diagnostic gold standard.

*We suggest Erect CXR as initial routine diagnostic assessment in case of acute abdomen from suspected free perforation of PU.*

*In case of negative AXR and/or erect CXR, we suggest CT scan as second level diagnostic tool since its higher sensitivity in detecting intra-abdominal free air.*

*In case of negative findings of free intra-abdominal air and persistent suspicion of PPU, we suggest adding oral water soluble contrast or via NGT.*

### Treatment

#### Non operative management

Crofts TJ et al. in 1989 conducted a prospective randomized trial comparing the outcome of nonoperative treatment with that of emergency surgery in patients with a clinical diagnosis of perforated peptic ulcer. Of the 83 patients entered in the study over a 13-month period, 40 were randomly assigned to conservative treatment, which consisted of resuscitation with intravenous fluids, institution of nasogastric suction, and intravenous administration of antibiotics and ranitidine. Eleven of these patients (28 percent) had no clinical improvement after 12 hours and required an operation. Two of the 11 had a perforated gastric carcinoma, and 1 had a perforated sigmoid carcinoma. The other 43 patients were assigned to immediate laparotomy and repair of the perforation. The overall mortality rates in the two groups were similar (two deaths in each, 5 percent), and did not differ significantly in the morbidity rates (40 percent in the surgical group and 50 percent in the nonsurgical group). They concluded that in patients with perforated peptic ulcer, an initial period of nonoperative treatment with careful observation may be safely allowed except in patients over 70 years old, and that the use of such an observation period can obviate the need for emergency surgery in more than 70 percent of patients [43].

Songne B et al. in 2004 conducted a prospective trial of 82 consecutive patients, with diagnosis of perforated peptic ulcer. They were initially treated with non-operative procedure (nasogastric suction and intravenous administration of H2-blockers or proton-pomp inhibitors).

Clinical improvement was obtained with non-operative treatment in 54% of the patients (44/82). The overall mortality rate was 1%. In univariate analysis, significant predictive factors of failure of non-operative treatment were: size of pneumoperitoneum, heart beat >94 bpm, abdominal meteorism, pain at digital rectal exam, and age >59 years. In multivariate analysis, the significant factors were the size of pneumoperitoneum, heart beat, and abdominal meteorism. The association of these criteria: size of pneumoperitoneum > size of the first lumbar vertebra, heart beat >94 bpm, pain at digital rectal exam and age > 59 years, led to surgical treatment in all cases.

These results suggested that more than 50% of patients with perforated peptic ulcer respond to conservative treatment without surgery and that the association of few criteria (size of pneumoperitoneum, heart beat, pain at digital rectal exam and age) required emergency surgery [44].

In conclusion, the most important factor regarding the likely success or otherwise of non-operative management of a perforated peptic ulcer is whether the ulcer has sealed. This can be shown by gastrografin contrast study. In the authors experience if there is free leak of contrast from the ulcer, then surgery is needed. If the ulcer has sealed itself by adherent omentum etc., then non-operative treatment is indicated provided the patient does not have peritonitis or severe sepsis. Percutaneous drainage of collections may be needed later.

There is anecdotal evidence that gastric ulcers are less likely to seal spontaneously and also can be malignant therefore non-operative treatment of perforated gastric ulcers should be approached with caution.

*In the last 10 years we have not found in the literature any study recommending a conservative approach to PPU.*

*Nonetheless we recommend operative treatment of any PPU with pneumoperitoneum and signs of peritonitis.*

*We suggest that an initial trial of non-operative management may be suitable in stable non-peritonitic and not severely septic patients with PPU in abscence of significant pneumoperitoneum (i.e. small confined perforation with limited extraluminal air amount) as long as an upper GI contrast study has shown that the ulcer perforation has sealed and there no free extraluminal leak of contrast.*

## Surgery

### Open surgery vs laparoscopy

The number of patients who needed surgical intervention for complications of peptic ulcer, such as perforation, remained relatively unchanged [45,46].

Limiting surgical delay is of paramount importance in treating patients with PPU. In fact from the Danish Clinical Register of Emergency Surgery, a cohort study including 2668 patients showed that every hour of delay from admission to surgery was associated with an adjusted 2 · 4 per cent decreased probability of survival compared with the previous hour [47].

Perforated peptic ulcer disease is a common abdominal disease and laparoscopic surgery has changed the way such emergencies are managed. Perforated peptic ulcer disease is a condition for which the laparoscopic approach has significant attractions. Laparoscopy allows the confirmation of the diagnosis and furthermore allows the identification of the position, site, and size of the ulcer [27,48,49]. The procedure also allows closure of the perforation and adequate peritoneal toilette without the need for a large abdominal incision.

In the rare occurrence of large perforation with a severe contamination with food debris that can not be adequately removed laparoscopically, conversion may be required for complete peritoneal toilette. In such cases the perforation may be extensive and a resectional surgery may be needed.

Evidence for laparoscopic repair is equivocal [50]. In available evidence, the results after laparoscopic repair are not clinically different from open surgery, and no difference is found in abdominal septic complications, pulmonary complications, or abdominal collections [50]. The first randomized trial comparing laparoscopic and open repair of perforated peptic ulcer showed that the total operative time for laparoscopic repair was significantly increased but did result in a reduced requirement for postoperative analgesia [50]. However, in the same study there was no significant difference found in NG tube drainage, intravenous fluid usage, hospital stay, and return to normal diet [51]. More recent randomized, controlled trials have shown that laparoscopic repair is associated with shorter operative time, decreased postoperative abdominal drain use, reduced analgesic requirement, reduced hospital stay, earlier return to normal diet, and reduced morbidity [27]. Laparoscopic repair allows a earlier removal of the abdominal drain, NG tube, and an earlier return to normal diet and mobilization. Even in recent studies, authors have noted an increased operative time [52]; however, a recent study show, with experience, the time taken for laparoscopic repair can be comparable to open repair. Previous studies have shown a suture leak rate of 7% with laparoscopic repair; however, recent study demonstrate that this can be completely abolished and can be superior to open surgery, for which a leak rate of 2% has been reported [52,53]. In addition, the decrease in tissue dissection and the lack of large abdominal incision reduced the amount of opiate analgesia needed by patients. Lau et al. [51] showed similar results in 100 patients, in whom there was a reduced requirement for opiate analgesia. In contrast to previous studies, there’s a significant decrease in hospital stay in patient who underwent laparoscopic surgery [54] as well as a reduction in overall morbidity. Many authors have concluded that both open and laparoscopic repair of peptic ulcer are both effective treatments [52].

Some authors state that laparoscopy is more dangerous in a situation of prolonged peritonitis [55]. This is supported by the finding that pneumonia occurred more often in the laparoscopy group, although the duration of perforation was similar in both groups [55]. Experimental animal studies [56,57] have revealed that the increased intra-abdominal pressure of carbon dioxide pneumoperitoneum is associated with an increased risk of bacteraemia and sepsis when the duration of peritonitis exceeds 12 h 27. Pneumonia may also be caused by increased bacterial translocation from the peritoneal cavity into the bloodstream, but there is no evidence to support this concept from clinical studies [58]. There is not yet sufficient information about the outcome after open and laparoscopic repair in high-risk patients. Although risk levels (for example Boey score, Acute Physiology And Chronic Health Evaluation II) for perforated peptic ulcer affect the outcome after both open and laparoscopic repair, any outcome might still be improved by taking (or avoiding) one or other of the interventions. Some surgical centres [59] have suggested choosing the more familiar open repair for high-risk patients, although there is no hard evidence that this is necessarily the better option. Lunevicius et al. suggest that laparoscopic repair is at least as safe and effective as open repair in terms of wound infection and mortality rates, and shorter hospital stays. The minimally invasive method is associated with a less painful recovery (balanced by a higher leak rate) and better cosmesis, fewer adhesions and incisional hernias, and better diagnostic potential. Patients with no Boey risk factors (prolonged perforation for more than 24 h, shock on admission and confounding medical conditions, defined as ASA grade III–IV) should benefit from laparoscopic repair [33].

Sanabria A. et al. in collaboration with the Cochrane library has made a review in 2010. They showed that there was a tendency to a decrease in septic intra-abdominal complications, surgical site infection, postoperative ileus, pulmonary complications and mortality with laparoscopic repair compared with open surgery, none of these were statistically significant. However, there was a tendency to an increase in the number of intra-abdominal abscesses and re-operations, but without statistical significance. This finding could be related to surgeon experience in laparoscopic surgery. It is not possible to draw any conclusions about suture dehiscence and incisional hernia with the two procedures [60]. Recently Guadagni et al. suggests that laparoscopic repair for PPU is feasible but skill in laparoscopic abdominal emergencies are required. Perforations 1.5 cm or larger, posterior duodenal ulcers should be considered the main risk factors for conversion [61].

Comparing laparoscopic versus open repair for PPU, Byrge N et al. has showed that in the laparoscopic group the rates of wound complications, organ space infections, prolonged ventilation, postoperative sepsis, return to the operating room, and mortality tended to be lower for the LA, although not significantly. Length of hospital stay was, however, significantly shorter for the laparoscopic repair. The authors concluded that laparoscopy is safe in mild to moderately ill patients with perforated peptic ulcer and may allow a reduced use of hospital resources [62].

*Laparoscopy allows the surgeon to explore and wash out the entire peritoneal cavity and it is therefore a powerful diagnostic tool. The benefits of less postoperative pain, shorter length of hospital stay and earlier return to work after laparoscopic surgery for perforated peptic ulcer may offset the costs needed for performing laparoscopic repair.*

*Laparoscopic repair also offers the advantage of better cosmesis.*

*We recommend laparoscopic approach to hemodynamically stable patients with free air at X-ray and/or CT for diagnostic purposes.*

*We suggest laparoscopic repair of PPU in stable patients with PPU <5 mm in size and in presence of appropriate laparoscopic skills.*

*We recommend laparoscopy for achieving a better intraperitoneal lavage, even in presence of diffuse peritonitis.*

*We suggest that laparoscopy may improve patients’ outcome with significantly lower morbidity.*

*We recommend open surgery in presence of septic shock or in patients with absolute contraindications for pneumoperitoneum.*

***We suggest open surgery in presence of perforated and bleeding peptic ulcers, unless in stable patients with minor bleeding and in presence of advanced laparoscpic suturing skills (Additional file*** ***1*** ***: Video 1).***

***We suggest use of intra-operative methylene blue via NG tube for precise localization of microscopic perforations (Additional file*** ***2*** ***: Video 2).***

### Primary repair vs sutureless

Laparoscopic sutureless repair was shown to take a significantly shorter time than laparoscopic suture repair. Laparoscopic sutureless repair has the advantage over laparoscopic suture repair because is technically much less demanding. The technique can be easily performed by those who have limited experience with laparoscopic surgery [63].

It is arguable if there are standard laparoscopic procedures to treat PPU. Sutureless repair was once considered as safe as suture repair [63] but it carried extra-costs such as the use of fibrin glue. Although the rationale of this sutureless technique was to simplify the procedure and shorten operative time, it did not gain wide acceptance owing to its high leakage rate as compared to suture repair (16–6%) [64]. Siu et al. [65] proposed a technique of closing the ulcer with a single stitch plus omental patch for small perforations (i.e. \10 mm). They obtained satisfactory results with a conversion rate of only 7.4% [66,27]. Song et al. [67] further simplified the method by suturing the perforation without knotting followed by tying the suture over an omental patch. Although simple and effective by avoiding applying suture on fragile edge, the draw back was that no further rescue maneuver could be made if the single stitch was tied without good security. Ates et al. [68] compared the results of laparoscopic simple closure without omental patch with that of conventional open repair in patients with small perforated duodenal ulcer and prove that is was as safe and as effective. On the other hand, Turner et al. [69] reported that suture without an omental patch would result in a significantly higher mortality rate than with a patch. However, most cases in their series were perforated gastric ulcers instead of juxta-pyloric perforation. Finally, Lunevicius et al. [70] reviewed 13 prospective and 12 retrospective studies and concluded that repair method should best be judged by the properties of the ulcer edge. In short, although it seems that no single method is considered being the standard, the literature showed that there were no differences between these two most common adopted procedures in terms of postoperative recovery and incidence of surgical complications. To summarize, laparoscopic simple closure alone without adding an omental patch is a safe procedure for juxtapyloric perforation in low risk patients. In terms of leakage rate and surgical outcome, the manoeuver to cover an omental patch on the repaired PPU did not show any additional advantage [71].

*We suggest that Laparoscopic sutureless repair may be a viable option in presence of limited laparoscopic experience, only in presence of small size perforations (i.e. microscopic or <2 mm perforations) without significant peritoneal contamination and for low risk patients.*

***We recommend primary repair in case of perforated peptic ulcer larger than 5 mm and smaller than 2 cm (Additional file*** ***3*** ***: Video 3).***

***We suggest routine use omental patch to further protect the suture line (see Additional file*** ***3*** ***: Video 3).***

*We recommend avoiding use of glue as only method of closure of PPU.*

*We suggest use of glue only as an adjunctive measure to protect suture line or the omental patch.*

*We suggest avoiding use of glue because of increased costs and risks of complications if serious doubts exist on the efficacy of primary closure.*

*We suggest conversion to open procedure if the primary repair is deemed to be done not efficaciously.*

### Resectional surgery

The resection surgery is a viable option for giant peptic ulcers, commonly defined as having a diameter greater than 2 cm. These lesions have a higher risk of perforation. In gastric lesions, although the risk of malignancy is less than historically predicted, the incidence is still around 10% [72,73]. There are no specific surgical treatment recommendations since the site of perforation and the secondary effects on the surrounding anatomical structures must direct the necessary interventions. These patients are also frequently in septic shock upon presentation when the amount of peritoneal spillage is large. This factor alone should significantly influence the choice of operative intervention. Giant gastric ulcers are most commonly located on the lesser curvature and will often require an antrectomy and reconstruction. For perforated giant duodenal ulcers, the defect is often too large to perform a primary repair. Leak rates of up to 12% have been reported from attempted closure with an omental patch procedure [74]. The proximity of the defect and its relation to the common bile duct and ampulla of Vater must also be thoroughly investigated. Intraoperative cholangiography may even be necessary to verify common bile duct anatomy. There are several different procedures that have been described for duodenal defects such as a jejunal serosal patch, tube duodenostomy, and several variations of omental plugs antrectomy with diversion is the classic and most commonly described intervention, if the ampullary region is not involved. Affected patients are often in extremis at the time of presentation, and therefore a damage control procedure will likely be the safest and most appropriate operation for the patient. An antrectomy, with resection of the duodenal defect for duodenal ulcers proximal to the ampulla, will allow a definitive control of the spillage. Depending upon the location of the duodenal defect, closure and diversion via antrectomy may be the safest method for damage control.

The proximal gastric remnant should be decompressed with a nasogastric tube placed intraoperatively with verification of its correct position. Anastomoses should be avoided in presence of hypotension or hemodynamic instability, especially if the patient requires vasopressors. After copious abdominal irrigation, a temporary abdominal closure device can be placed. The patient can then be resuscitated appropriately in the ICU. The surgeon can return to the OR for re-exploration, restoration of continuity, possible vagotomy, and closure of the abdomen once the patient is hemodynamically stable [75].

***We suggest resectional surgery in case of perforated peptic ulcer larger than 2 cm (Additional file*** ***4*** ***: Video 4)***

***We suggest resectional surgery in presence of malignant perforated ulcers or high risk of malignancy (e.g. large ulcers, endoscopic features of malignancy, presence of secondary lesions or suspected metastases, etc.) (Additional file*** ***4*** ***: Video 4).***

*We suggest resectional surgery in presence of concomitant significant bleeding or stricture.*

*We suggest use of techniques such as jejunal serosal patch or Roux en-Y duodenojejunostomy or pyloric exclusion to protect the duodenal suture line, in case of large post-bulbar duodenal defects not amenable to resection (i.e. close to or below the ampulla).*

*Whenever possible (i.e. stable patient), in case of repair of large duodenal ulcer, we suggest to perform a cholecistectomy for external bile drainage (e.g. via trans-cystic tube).*

*We suggest duodenostomy (e.g. over Petzer tube) only as an extreme option, in presence of giant duodenal ulcers with severe tissue inflammation and when mobilization of the duodenum is not possible and the patient is in severe septic shock/hemodynamic instability.*

## Other techniques and future developments

### Self-expandable metal stents

Primary stenting and drainage has been shown to be an effective and safe way to treat esophageal perforations or anastomotic leaks after gastric bypass surgery.

M. Bergstrom et al. present a case series of eight patients with perforated duodenal ulcers treated with covered self-expandable metal stents (SEMS).

Two patients received their stents because of postoperative leakage after initial traditional surgical closure. Six patients had SEMS placed as primary treatment due to co-morbidities or technical surgical difficulties. Endoscopy and stent treatment in these six patients was performed at a median of 3 days (range, 0–7 days) after initial symptoms. Six patients had percutaneous abdominal drainage. Early oral intake, 0–7 days after stent placement, was possible. All patients except one recovered without complications and were discharged 9–36 days after stent placement. This study indicates that in cases where surgical closure will be difficult, gastroscopy with stent placement can be performed during the laparoscopy, followed by laparoscopic drain placement. In patients with severe co-morbidity or delayed diagnosis, gastroscopy and stent placement followed by radiologically guided drain placement can be an alternative to conservative treatment [76].

### Natural orifice transluminal endoscopic surgery (NOTES)

A NOTES approach may reduce the physiologic impact of therapeutic intervention after peptic ulcer perforation and provide a technically less challenging procedure. Experimental data suggest that the NOTES repair may be possible with lower intraabdominal pressure [77]. Preclinical trials of endoscopic omental patch closures for upper gastrointestinal viscus perforations have been published [78]. A retrospective review suggested that up to 50% of patients presenting with perforated ulcer might be candidates for a NOTES repair [79].

Bingener et al. [80] present a pilot clinical study evaluating the feasibility of endoscopic transluminal omental patch closure for perforated peptic ulcers, with the hypothesis that the technique will be successful at closing ulcer perforations, as evidenced by intraoperative leak test and post operative water-soluble contrast studies.

After induction of general anesthesia, pneumoperitoneum (12–14 cm H2O) has been established using a periumbilical trocar in Hasson technique. This served to confirm the diagnosis of ulcer perforation and for surveillance of the endoscopic procedure. A standard diagnostic upper endoscope with CO2 insufflation has been introduced through the oropharynx into the stomach and duodenum. The site of perforation was identified and measured. The endoscope was carefully advanced through the perforation when possible. Once in the peritoneal cavity, the endoscopist proceeded with inspection and irrigation. A viable mobile piece of omentum was identified, and pulled intraluminally through the site of perforation. The omentum was then fixed to the mucosa of the luminal wall with several endoscopic clips. The falciform ligament was used if a suitable omental patch was not available. If the NOTES procedure was unsuccessful, either a laparoscopic or open omental patch repair was considered by the acute care surgical team [80].

Initial results from a laparoscopic-assisted NOTES approach for closure of perforated peptic ulcers appear promising and enable swift recovery in selected patients. This is especially important in elderly and/or immunocompromised patients. Technical details and patient selection criteria continue to evolve.

*We do not recommend NOTES approach for PPU treatment until further experience and clinical evidence is gained.*

## Diagnosis and treatment of bleeding peptic ulcer (Dr. M. Bassi MD)

### Introduction

Acute upper gastrointestinal bleeding (UGIB) is the most common gastroenterological emergency and has a considerable morbidity and mortality. Management strategies have changed dramatically over recent decades due to the introduction of acid suppressive therapy, especially proton pump inhibitors (PPIs), and endoscopic therapy.

The incidence rates of UGIB demonstrate a large geographic variation ranging from 48 to 160 cases per 100 000 population [81-84].

Possible explanations for the reported geographic variation in incidence are: differences in definition of UGIB in various studies, population characteristics, prevalence of ulcerogenic medication, in particular aspirin and nonsteroidal anti-inflammatory drugs (NSAIDs), and *Helicobacter pylori* (*H. pylori*) prevalence.

Some but not all time-trend studies have reported a significant decline in incidence of acute UGIB, especially peptic ulcer bleeding (PUB), in recent years. This decline is likely due to a combination of factors, including decreasing prevalence of gastric colonization with *H. pylori,* the use of eradication therapy in patients with ulcer disease, and the increased use of PPI therapy, both in general and in patients using aspirin and NSAIDs in particular [81,85].

At the same time, an increasing proportion of patients presenting with UGIB are older and a significant number of patients with UGIB consume NSAIDs and/or antiplatelet therapy to treat other medical comorbidities. Given these factors, UGIB continues to have a considerable impact with respect to patient morbidity and mortality, as well as health care resource utilization. The mortality rate of UGIB remains high somewhere between 7% and 14%. UGIB accounts for > 300 000 annual hospitalizations in the United States, with an estimated cost of $ 2.5 billion [86-88].

The majority of deaths do not directly result from exsanguination, but are related to poorly tolerated blood loss and resultant shock, aspiration, and therapeutic procedures. As such, mortality from UGIB is strongly associated with advanced age and presence of severe comorbidity. The risk of mortality increases with rebleeding, which is thus another major outcome parameter.

The incidence of rebleeding in patients with UGIB shows a wide range from 5% to more than 20%, depending on the aetiology of the bleeding and the timing of endoscopic therapy. There is strong evidence that the risk of rebleeding is highest in the initial period of admission, and a 24-h time frame for endoscopic therapy is internationally recommended as the optimal window of opportunity. Naturally, rebleeding must be prevented whenever possible [86,89].

PUB is the most common cause of acute UGIB, accounting for 31%-67% of all cases, followed by erosive disease, varices, oesophagitis, malignancies and Mallory-Weiss tears (Table 3) [81,83,90].

**Table 3:** Causes of upper gastrointestinal bleeding

	**%**
Peptic ulcer	31–67
Erosive	7–31
Variceal bleeding	4–20
Oesophagitis	3–12
Mallory-Weiss	4–8
Malignancies	2–8
Other	2–8

In the subgroup of patients with PUB, bleeding from duodenal ulcers is slightly more frequent than from gastric ulcers [91].

Emergency surgery for PUB has continued to decrease; in the UK, the rate of surgery dropped from 8% to 2% between 1993 and 2006. In the same period in the USA, admissions to hospital for peptic ulcer bleeding fell by 28,2%, the use of endoscopic treatment increased by 58,9%, and the rate of emergency surgery for PUB decreased by 21,9% [92-94].

### Initial assessment, resuscitation and risk-scores

A primary goal of the initial assessment is to determine whether the patient requires urgent intervention (e.g., endoscopic, surgical, transfusion) or can undergo delayed endoscopy or even be discharged to outpatient management.

Patients presenting with acute UGIB should be assessed promptly and resuscitated if needed. Volume should be replenished initially with crystalloid solutions.

In patients with ongoing blood loss, symptomatic anaemia, or those at increased risk of impaired tissue oxygenation (e.g., patients with chronic heart conditions), blood should be transfused. In haemodynamically stable patients who are not bleeding actively, the threshold of transfusion needs to be defined. International guidelines recommend a policy of transfusion to a haemoglobin concentration of 7 g/dL [86].

Coagulopathy at presentation is a major adverse prognostic factor. From the UK National Audit, coagulopathy defined by an international normalised ratio (INR) above 1,5 was present in 16,4% of patients and was associated with a 15% mortality rate [95].

Coagulopathy is also a marker for other comorbidites, such as chronic liver disease. Bleeding in these patients is often more severe, and coagulopathy should be corrected in those with active bleeding. The target INR has not been defined and is established by the patient’s indication for anticoagulation. A study showed that mild to moderate anticoagulation (INR 1,3–2,7) at endoscopy did not increase the risk of recurrent bleeding compared with an INR of less than 1,3 [96].

One small cohort study with a historical comparison showed that aggressive resuscitation including correction of coagulation (INR <1,8) led to lower mortality rates [97].

Although numerous factors from the patient history, physical examination, and initial tests have been examined for an association with a need for intervention, no single factor is sufficiently predictive of UGIB severity to be used for triage [98].

The most predictive individual factors are a history of malignancy, presentation with hematemesis, signs of hypovolemia including hypotension, tachycardia and shock, and a haemoglobin < 8 g/dL [99,100].

Some factors, such as a history of aspirin or NSAIDs use, may not be useful for immediate disposition but are still important to assess for future management (e.g., if PUB were the aetiology of UGIB, then NSAIDs use should be discontinued). Patients who have significant comorbidities may require admission regardless of the severity of the UGIB [98,101].

Several scoring systems have been created and/or validated for this purpose, including APACHE II, Forrest classification, Blatchford score, pre-endoscopic Rockall score. Some of these may be cumbersome (APACHE II) or require data not immediately available based on initial clinical assessment (the Rockall Scoring System, for instance, requires endoscopic data) and therefore may be of limited utility in the acute setting [87,102].

The Blatchford score and the pre-endoscopic Rockall score have been examined in several studies and may determine the need for urgent endoscopy (Table 4) [103,104].

**Table 4:** Comparison of Blatchford and Rockall risk scoring systems

**Risk factor**	**Blatchford Score**		**Pre endoscopic Rockfall score**	
	**Parameter**	**Score**	**Parameter**	**Score**
Age (yr)	-		60-79	1
	-		≥ 80	2
SBP (mmHg)	100-109	1	<100	2
	90-99	2	-	
	<90	3	-	
BPM	> 100	1	> 100 with SPB ≥ 100	1
Clinical presentation	Melena	1	-	
	Synocpe	2	-	
Comorbidity	Hepatic disease	2	CHF, IHD, major comorbidity	2
	Cardiac failure	2	Renal or liver failure, metastases	3
Blood urea (mg/dL)	18.2-22.3	2	-	
	22.4-27.9	3	-	
	28-69.9	4	-	
	≥ 70	6	-	
Hemoglobin g/dL	F: 10–11.9	1	-	
	M: 10–11.9	3	-	
	F/M: < 10	6	-	
			**Complete Rockfall score**	
Endoscopic diagnosis	-		Non malignant, non Mallory-Weiss	1
	-		Upper GI malignancy	2
Evidence of bleeding	-		Blood, adherent clot, active bleeding	2

M: Male; F: Female; SBP: Systolic blood pressure; CHF: Congestive heart failure; IHD: ischemic hearth disease.

The Blatchford score uses data on blood urea and haemoglobin levels, systolic blood pressure, pulse, presentation with melena, presentation with syncope, history of hepatic disease, and history of heart failure. A Blatchford score > 0 was 99% to 100% sensitive for identifying a severe bleed in 5 studies [103,105].

The specificity of the Blatchford scoring system is low (4%-44%), but clinically it is more important to be comfortable identifying all severe UGIB at the expense of admitting some patients with minor bleeding episodes. Patients found to have minor bleeding episodes typically may be discharged soon after endoscopy. Use of the Blatchford score may allow early discharge of 16% to 25% of all patients presenting with UGIB [103,105,106].

The use of a nasogastric tube remains controversial [98]; in theory, the presence of bright red blood *via* nasogastric aspirate suggests active UGIB and should prompt urgent to esophagogastroduodenoscopy (EGD).

The absence of blood on nasogastric aspirate, however, does not exclude the presence of a culprit UGIB source [81].

In a study by Aljebreen et al., 15% of patients with UGIB and clear or bilious nasogastric aspirate were ultimately found to have an underlying high risk lesion during EGD [100].

### Pharmacologic therapy prior to endoscopy

Early administration of intravenous PPIs in patients who present with signs of UGIB is reasonable. A Cochrane meta-analysis of six randomised controlled trials (n = 2223) noted a reduction in high-risk stigmata of bleeding (37,2% *vs.* 46,5%,) with early use of PPIs and a lower proportion of patients undergoing endoscopic therapy (8,6% *vs.* 11,7%).

The reduction in endoscopic treatment leads to early discharge in some patients with clean-based ulcers and low-risk stigmata and is cost saving.

However, the use of proton-pump inhibitors should not replace urgent endoscopy in patients with active bleeding [94,107].

A prokinetic drug given before endoscopy helps to empty stomach contents and improves viewing at endoscopy. These drugs are rarely used by endoscopists. Only five randomised trials and their pooled analysis have been published: three with the use of erythromycin and two with metoclopramide.

The use of these drugs reduces the need for a second endoscopic examination for diagnosis but no significant difference in other clinical outcomes was recorded [94,108].

At present, insufficient evidence exists to support the use of tranexamic acid in acute PUB [94].

### Endoscopic treatment

Endoscopy in patients with PUB is effective and is associated with a reduction in blood transfusion requirements and length of intensive care unit/total hospital stay [98,109].

The optimal timing for endoscopy in PUB remains under debate [81].

In appropriate settings, endoscopy can be used to assess the need for inpatient admission.

Several studies have demonstrated that hemodynamically stable patients who are evaluated for UGIB with upper endoscopy and subsequently found to have low-risk stigmata for recurrent bleeding can be safely discharged and followed as outpatients [110,111].

Patients with unstable haemodynamics and active haematemesis should be offered urgent endoscopy with a view to haemostasis. Patients who are stable after initial resuscitation generally undergo endoscopy the next morning. Evidence for the use of early endoscopy (generally defined by endoscopy within 24 h) came from cohort studies and their meta-analysis and results in significantly reduction of the hospital stay and improvement of the outcome [86,94,112].

However, although emergency endoscopy should be considered in patients with severe bleeding, very early endoscopy (<12 h) has so far not been shown to provide additional benefit in terms of reduction of rebleeding, surgery and mortality, compared with later endoscopy (within 24 h) [113-115].

The Forrest classification is often used to distinguish endoscopic appearances of bleeding ulcers (Ia spurting active bleeding; Ib oozing active bleeding; IIa visible vessel; IIb adherent clot; IIc flat pigmented spot; III ulcer with a clean base) [116].

*In PUB, patients with active bleeding ulcers or a non-bleeding visible vessel in an ulcer bed are at highest risk of re-bleeding and therefore need prompt endoscopic hemostatic therapy.*

Patients with low-risk stigmata (clean-based ulcer or a pigmented spot in ulcer bed) do not require endoscopic therapy [81].

Two small randomised trials, and a meta-analysis suggested that a clot should be removed in search of an artery and, when it is present, endoscopic treatment should be given, although the management of peptic ulcers with overlying adherent clots that are resistant to removal by irrigation is still controversial [98,117-119].

Endoscopic treatment can be divided into injection (including epinephrine, sclerosants and even normal saline solution), thermal (including monopolar or bipolar cautery and argon plasma coagulation) and mechanical methods (including hemoclips).

Often, the choice of which endoscopic therapy employ is based on local preference and expertise.

Injection of diluted epinephrine alone is now judged to be inadequate [94].

Cushions of fluid injected into the submucosa compress the artery to stop or slow down bleeding and allow a clear view of the artery. A second modality should be added to induce thrombosis of the artery.

Calvet et al. pooled the results of 16 randomised controlled trials that compared injection of diluted adrenaline alone with injection followed by a second modality, and showed that combination treatment led to substantial reductions in rate of recurrent bleeding (risk reduction from 18,4% to 10,6%), surgery (from 11,3% to 7,6%) and mortality (from 5,1% to 2,6%) [120].

The investigators also compared studies with or without second look endoscopies after initial endoscopic treatment. Rebleeding was higher in the group given adrenaline injection alone than in the combination treatment group (15,7% vs. 11,4%).

Two other meta-analyses that summarised studies of monotherapies versus dual therapies also concluded that a second modality should be added to injection treatment [108,121].

The observation suggested that if combination treatment had been instituted at index endoscopy, a second look endoscopy would have been unnecessary, so routine second look endoscopy after initial endoscopic haemostasis is not recommended [122].

A new promising endoscopic application is the use of a chemical compound which, when sprayed as nanopowder on active bleeding, can lead to immediate hemostasis, with coverage of the bleeding ulcer with a powder layer. In a pilot study of 15 patients with active PUB treated with this nanopowder, immediate hemostasis was achieved in 93%, and one patient had recurrent bleeding. No adverse events were reported during the follow-up. Further studies with this product are ongoing [123].

*Early endoscopy (within 24 h) in PUB results in significantly reduction of the hospital stay and improvement of the outcome. Dual endoscopic therapy, rather than monotherapy, led to substantial reductions in rate of recurrent bleeding, surgery and mortality .*

### Postendoscopic management

Pharmacotherapy plays a second major role in the treatment of PUB. PPIs can be administered orally or intravenously depending on the rebleeding risk.

In a randomized placebo-controlled trial of 767 PUB patients treated with endoscopic therapy because of high-risk stigmata, high-dose intravenous PPIs (80 mg esomeprazole bolus *plus* 8 mg/h continuous infusion for 72 h) significantly reduced rebleeding (5.9% *vs.* 10.3%, *P* = 0.03) and the need for endoscopic retreatment [124].

Similar results were found by meta-analysis; high-dose intravenous PPIs after endoscopic therapy significantly reduced rebleeding, need for surgery and mortality compared with placebo/no therapy [125].

PPIs are recommended for 6–8 weeks following UGIB and/or endoscopic treatment of PUD to allow mucosal healing [126].

Once mucosal healing has been achieved, how long it should last the PPIs use is still controversial.

Studies have shown that in patients who have PUD complicated by bleeding, there is a 33% risk of rebleeding in 1–2 years. Furthermore, there is a 40%-50% rebleeding risk over the subsequent 10 years following the initial episode of bleeding [100].

Randomized prospective trials have demonstrated a benefit to long-term acid-suppression therapy in two settings: chronic NSAID users and *H. pylori*-infected patients [127].

Testing for *H. pylori* is recommended in all patients with PUB.

This should be followed by eradication therapy for those who are *H. pylori*-positive, with subsequent assessment of the effect of this therapy, and renewed treatment in those in whom eradication fails [86].

*High-dose continuous intravenous PPIs is recommended in patients with PUB and high-risk stigmata.*

### Continued and recurrent bleeding

Despite adequate initial endoscopic therapy, recurrent UGIB can occur in up to 24% of high-risk patients [98].

Mortality after a surgical salvage in the recent UK National Audit was 29% [128].

Large ulcers located in the posterior bulbar duodenum and lesser curvature of stomach can erode into the gastroduodenal or the left gastric artery, respectively, which are predictive of endoscopic treatment failure.

These ulcers often occur in elderly patients who present with a major bleed in shock and low initial haemoglobin concentrations [129].

Patients with massive bleeding who do not respond to endoscopy are often shifted to surgical treatment.

Angiographic embolization is an alternative when its expertise is immediately available.

Loffroy et al. summarised outcomes in ten case series of 75 patients treated with embolization. The rate of clinical success, rebleeding, and mortality rate was 75%, 25%, and 25%, respectively [130].

In retrospectives comparisons of angiographic embolization versus surgery, in patients with PUB who do not respond to endoscopic haemostatic attempts, angiographic embolization was associated with reduced treatment-related complications (20–54% *vs.* 37–68%). Mortality after either treatment was similar (3–30% *vs.* 14–30%) [131-133].

A randomised controlled trial compared surgery with further endoscopic treatment for rebleeding. In 75% of these patients, further endoscopic treatment led to durable haemostasis. Patients randomly allocated to surgery had substantially more postoperative complications.

However, a sub-group analysis suggested that ulcers larger than 2 cm and a major rebleeding with hypotension were factors that predicted failure in further endoscopic attempts; thus, in these patients, surgery or angiographic embolization should be immediately available if repeated endoscopic treatment fails [134].

A recent study suggests transcatheter superselective angioembolization, with reembolization if necessary, is an effective rescue treatment modality for hemodynamically unstable patients with active gastrointestinal hemorrhage and is a reasonable management option. Twenty percent of patients will fail superselective angioembolization and require additional intervention. Ischemic complications are extremely rare [135].

For patients with intractable ulcer bleeding, Schroeder et al. from the analysis of large database (ACS-NSQIP) have found that the surgical procedure of vagotomy/drainage is associated with significantly lower mortality than just with simple local ulcer oversew. They futher suggest that vagotomy/drainage is preferred to local procedures alone for the surgical management of patients with bleeding peptic ulcer disease requiring emergency operation for intractable bleeding ulcers [136].

Open surgery is recommended when endoscopic treatments failed and there is evidence of ongoing bleeding +/− hemodynamic instability. The surgeon may not know preoperatively where the bleeding comes from and intraoperative endoscopic guidance may be helpful. A retractor that elevates the sternum might be needed (the so called Goligher sternal-lifting retractor) and sometimes is necessary to excise the xiphisterum. Then, after defusing the spleen, the oesophagus should be taped to enable control of stomach. In case of bleeding gastric ulcer (GUs), anterior gastrotomy can be easily performed. In case of bleeding duodenal ulcer (DUs) it might be needed to perform a duodenotomy and open across D1 and pylorus, longitudinally.

Bleeding GUs should be resected (even just a local resection) or at least biopsied for the possibility of neoplasms. Most of DUs arriving to surgery for persistent bleeding are usually big and posterior lesions and the bleeding is often from gastro-duodenal artery. Via duodenotomy, the bleeding vessel can be seen on the floor of the ulcer and can be rapidly oversewn; then the duodenotomy is closed normally with horizontal sutures to avoid stenosis and without need of routine pyloroplasty.

A Billoth-1 resection with distal gastrectomy might be needed if D1 is fully shattered by a large duodenal ulcer.

*Surgical hemostasis or angiographic embolization (where readily available) should be performed only after endoscopic failure.*

*Open surgery is recommended when endoscopic treatments failed and there is evidence of ongoing bleeding +/− hemodynamic instability.*

### Peptic ulcer bleeding in patients receiving anti-thrombotic therapy

Patients on antiplatelets or anticoagulant therapy with acute UGIB represent a major challenge and need to be managed on a individual basis and the best way to treat patients on antithrombotic drugs with acute UGIB is clinically challenging.

These patients are of course at high risk of thromboembolism because of their underlying cardiovascular illness.

However, discontinuation of anti-thrombotic therapy may be necessary to control bleeding or prevent rebleeding.

A multidisciplinary and individualized evaluation is needed to decide either to stop or to resume anti-thrombotic, balancing thromboembolic risk against the risk of bleeding.

In a randomised trial of continuous versus discontinued aspirin treatment in patients with PUB and high cardiothrombotic risks, those receiving continuous aspirin had a twofold increased risk of early recurrent bleeding (10,3% *vs.* 5,4% at day 30) but a tenfold reduced risk of mortality (1,3% *vs.* 10,3% at 8 weeks) compared with those remained without aspirin [137].

In patients at low risk of recurrent bleeding, aspirin can be resumed the after-bleeding morning.

The antiplatelet effect of aspirin lasts for about 5 days and the risk of early recurrent bleeding is high in the first 3 days; thus, in high-risk cardiovascular patients, it might be reasonable to resume aspirin on fourth day after bleeding to minimise both bleeding and thrombotic risks [94].

Patients on dual antiplatelet treatment (e.g. aspiring and clopidogrel), especially after recent placement of drug-eluting coronary stents, are at high risk of thrombosis. In patients at low risk of recurrent bleeding, dual antiplatelet treatment should be continued.

In those at high risk, cessation of both antiplatelet drugs should be avoided, given the very high risk of stent occlusion [138].

In high-risk patients, after endoscopic control of bleeding, high-dose PPIs infusion and temporarily withholding of clopidogrel is recommended.

Early resumption of clopidogrel should be considered in patients who had stent placement within 4 weeks, left main stem disease, and known coronary artery dissection [94].

Major gastrointestinal bleeding is often associated with anticoagulant therapy.

Rapid correction of the coagulopathy is recommended.

Intravenous vitamin K will reverse the coagulopathy due to warfarin, but its full effect can take up to 24 hours.

Prothrombin complex concentrates rapidly reverse coagulopathy, and this treatment is preferred over fresh frozen plasma, especially in patients with cardiac and renal failure who poorly tolerate fluid overload [139].

If anticoagulant therapy has been prescribed there is a high-probability that this patients are at high risk of thrombosis; treatment with low-molecular-weight or unfractionated heparin should be considered in almost all cases [94]. However the treatment with unfractionated heparin in the initial stage can be more easily controlled than low molecolar weight heparin.

Bleeding in patients treated with new oral anticoagulants (NOACs), which include dabigatran, rivaroxaban, apixaban, and edoxaban, represents an extreme challenge. Currently no antidote exists to reverse the effects of these drugs. Specific antidotes for the reversal of the anticoagulant effect of these drugs, such as monoclonal antibodies against the direct thrombin inhibitor dabigatran or recombinant Xa-analog in the case of factor Xa inhibitors, are still being investigated in early clinical trials. In certain situations, as in case of emergency surgery or life-threatening major bleeding, a rapid reversal strategy is needed. Several non-specific prohemostatic agents or coagulation factor concentrates have been suggested as potential candidates for the reversal of NOACs. Activated prothrombin complex concentrate seems promising for the reversal of dabigatran, while non-activated prothrombin complex concentrates have potential for the reversal of anti-factor Xa [140]. In such cases a consultation between critical care speciliast, haematologist and a nephrologists is recommended.

## 

This article contains supplemental online multimedia material.

## Competing interests

The authors declare that they have no competing interests.

## Authors’ contributions

Study conception and design: SDB, NS, FC, LA, VC, EJ. Acquisition of data: NS, MB, SDS, VC. Analysis and interpretation of data: MB, SDS, NS, VC. Drafting of manuscript: NS, MB, SDS. Critical revision: SDS, MB, NS, MM, FF, CF, LA, SG, MS, FC, NN, MS, GT, FC, VC, EJ. Final approval of the final version. SDS, MB, NS, MM, FF, CF, LA, SG, MS, FC, NN, MS, GT, FC, VC, EJ. All authors read and approved the final manuscript.

## Supplementary Material

Additional file 1: Video 1 Laparoscopic suture and repair of perforated and bleeding ulcer in a patient hemodynamically stable; Operating Surgeon Dr. Salomone Di Saverio MD. Click here for file

Additional file 2: Video 2 Difficult localization of a small PPU: use of Methylene Blue via NGT for localization; Operating Surgeon Dr. Salomone Di Saverio MD. Click here for file

Additional file 3: Video 3 Technique of laparoscopic primary suture and repair of PPU larger than 1 cm; Operating Surgeon Dr. Salomone Di Saverio MD. Click here for file

Additional file 4: Video 4 Laparoscopic finding of a very large malignant perforated ulcer of the posterior gastric wall: an indication for conversion and open total gastrectomy; Operating Surgeon Dr. Salomone Di Saverio MD. Click here for file
